# Overexpression of *CmWRKY8*-1–*VP64* Fusion Protein Reduces Resistance in Response to *Fusarium oxysporum* by Modulating the Salicylic Acid Signaling Pathway in *Chrysanthemum morifolium*

**DOI:** 10.3390/ijms24043499

**Published:** 2023-02-09

**Authors:** Weihao Miao, Lijiao Ge, Yuean Wang, Song Li, Daojin Sun, Ye Liu, Zhiyong Guan, Sumei Chen, Weimin Fang, Fadi Chen, Shuang Zhao

**Affiliations:** 1 College of Horticulture, Nanjing Agricultural University, Nanjing 210095, China; 2 Key Laboratory of Landscaping, Ministry of Agriculture and Rural Affairs, Nanjing 210095, China

**Keywords:** *Chrysanthemum morifolium*, *Fusarium oxysporum*, RNA-seq, salicylic acid, VP64, WRKY

## Abstract

Chrysanthemum Fusarium wilt, caused by the pathogenic fungus *Fusarium oxysporum*, severely reduces ornamental quality and yields. WRKY transcription factors are extensively involved in regulating disease resistance pathways in a variety of plants; however, it is unclear how members of this family regulate the defense against *Fusarium* wilt in chrysanthemums. In this study, we characterized the WRKY family gene *CmWRKY8-1* from the chrysanthemum cultivar ‘Jinba’, which is localized to the nucleus and has no transcriptional activity. We obtained *CmWRKY8-1* transgenic chrysanthemum lines overexpressing the *CmWRKY8-1-VP64* fusion protein that showed less resistance to *F. oxysporum*. Compared to Wild Type (WT) lines, *CmWRKY8-1* transgenic lines had lower endogenous salicylic acid (SA) content and expressed levels of SA-related genes. RNA-Seq analysis of the WT and *CmWRKY8-1-VP64* transgenic lines revealed some differentially expressed genes (DEGs) involved in the SA signaling pathway, such as *PAL*, *AIM1*, *NPR1*, and *EDS1*. Based on Gene Ontology (GO) enrichment analysis, the SA-associated pathways were enriched. Our results showed that *CmWRKY8-1-VP64* transgenic lines reduced the resistance to *F. oxysporum* by regulating the expression of genes related to the SA signaling pathway. This study demonstrated the role of *CmWRKY8-1* in response to *F. oxysporum*, which provides a basis for revealing the molecular regulatory mechanism of the WRKY response to *F. oxysporum* infestation in chrysanthemum.

## 1. Introduction

*Fusarium* wilt is a severe soil-borne disease that causes yellowing and wilting of plant leaves by damaging their vascular bundles. *Fusarium oxysporum* is the culprit of wilt disease, which usually invades the roots and multiplies the vascular bundles. *F. oxysporum* can block the vascular bundle and water cannot be transported, eventually leading to plant death [1,2,3]. The plant defense against pathogens relies on the natural immune system of plants. Pathogen-associated molecular pattern (PAMP)-triggered immunity (PTI) is triggered when PAMPs are sensed by pattern recognition receptors (PRRs) located on the surface of plant cells [4]. Intracellular nucleotide-binding leucine-rich repeat (NB-LRR) sensing specific effectors of pathogens can, in turn, trigger effector-triggered immunity (ETI) [5]. Among the transcription factors studied, WRKY transcription factors play an indispensable role in PTI and ETI immune pathways.

WRKY transcription factors are a family of transcription factors that are unique to plants and are widely distributed. WRKY transcription factors are widely involved in the regulation of plant growth, development, senescence, organ synthesis, and various hormone-mediated signaling pathways [6]. More importantly, WRKY transcription factors play an essential regulatory role in plant stress resistance pathways in response to biotic and abiotic stresses [7,8]. WRKY is named after its highly conserved WRKY structural domain, which consists of approximately 60 amino acid residues. The N-terminal end of the WRKY structural domain is a highly conserved heptapeptide sequence WRKYGQK, whereas the C-terminal end of the WRKY structural domain is a conserved C2H2 (CX4-5CX22-23HX1H) or C2HC (C-X7-C-X23-HX1-C)-type zinc finger structure [9]. WRKY transcription factors can be classified as I, II, and III based on the number of structural domains and the characteristics of the zinc finger structure [10]. In *Arabidopsis*, almost all class III WRKY transcription factors respond to biotic stress, suggesting that class III WRKY transcription factors may have evolved under biotic stress [11]. WRKY proteins can specifically recognize the W-box element (TTGACT/C) and regulate the expression of downstream genes [12]. In recent years there have been an increasing number of studies on the involvement of WRKY in plant disease resistance. In *Arabidopsis*, *AtWRKY8* and *AtWRKY33* have enhanced resistance to *Botrytis cinerea*, whereas *AtWRKY57* increases the sensitivity to *B. cinerea* [13,14,15]. WRKY transcription factors regulate plant defense systems through complex defense networks. For example, *CsWRKY25* can improve resistance to *Penicillium digitatum* by regulating ROS production and PR gene expression in citrus [16]. *CaWRKY40* interacts with *CaMPK9* to enhance the stability of *CaWRKY40*, and *CaWRKY40* positively regulated the transcription of *CaWRKY33* to improve resistance to *F. oxysporum* in chickpea [17]. Similarly, in chrysanthemums, *CmWRKY53* may reduce aphid resistance by regulating the production of secondary metabolites [18]. In addition, WRKY transcription factors are involved in phytohormone-mediated signaling pathways by regulating the anabolism of phytohormones, thereby regulating plant responses to biotic stress. Among plant hormones, salicylic acid (SA) usually plays an important regulatory role when plants are under biological stress [19]. More recently, *CmWRKY15-1* has been shown to enhance resistance to *Puccinia horiana Henn* via the SA signaling pathway in chrysanthemum [20], and *MdWRKY15* can bind to the W-box on the downstream *MdICS1* promoter, thus enhancing the accumulation of endogenous SA and improving apple resistance to *Botryosphaeria dothidea* [21].

Genetic engineering techniques have become essential tools for studying plant transcription factors. The fusion of dCas9 with a transcriptional activation domain enables the upregulation of target gene expression and is referred to as CRISPR activation. The VP64 fusion of the TAD tetramer of VP16 with the dCas9 protein is called dCas9-VP64. This complex is often capable of recruiting regulatory transcription factors. In mammals, dCas9-VP64 is often used to activate target genes. dCas9-VP64 consists of a C-terminal fusion of the VP64 activation domain of the dCas9 protein. This protein binds to the gene promoter regions mediated by gRNA and binds to PoIII through the VP64 activation region to activate gene transcription [22,23,24]. The dCas9-VP64 fusion protein can be used to activate not only reporter genes, but also to silence endogenous genes or up-regulate the expression of genes that are already activated, and has a wide range of applications. In recent years, dCas9-VP64 has been used in various plants. The role of the dCas9-VP64 system in UDP-glucose flavonoid glycosyltransferase gene activation is approximately 1.6-to 5.6-fold higher in grapes [25]. Rice can improve grain yield by delaying flowering when OsMYB1R1 is overexpressed with VP64 fusion protein [26]. However, dCas9-VP64 has been less studied with regard to disease resistance in chrysanthemums.

Chrysanthemums are among the top ten traditional flowers in China and one of the world’s four most famous cut flowers. Chrysanthemums have a profound history and culture of cultivation and are widely used in floral ornamental and medicinal cultivation [27]. Although the role of WRKY transcription factors in plant resistance mechanisms has been studied extensively, it is unclear whether members of the WRKY family in chrysanthemum contribute to the response to *F. oxysporum* infection. In the previous study of 15 WRKY transcription factors in chrysanthemum, Song found that *CmWRKY8* (KC615362) responded to the infestation of *F. oxysporum*, and it was inferred that *CmWRKY8* might be related to the resistance of chrysanthemum regarding *F. oxysporum* [28]. To test this hypothesis, we isolated and characterized *CmWRKY8-1* and studied its function in response to *F. oxysporum* using transgenic technology. Finally, we observed that *CmWRKY8-1* responds to *F. oxysporum* infestation by regulating the SA signaling pathway in chrysanthemum.

## 2. Results

### 2.1. Isolation and Sequence Analysis of CmWRKY8-1

**Figure 1:**
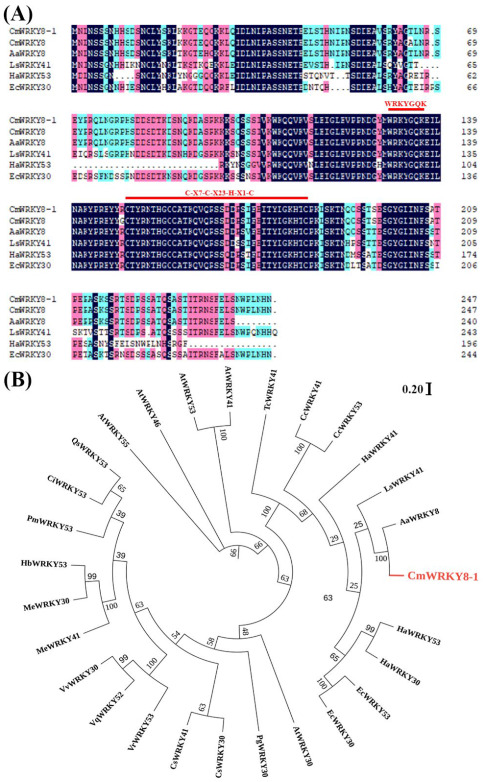
CmWRKY8-1 homologous proteins alignment and phylogenetic tree construction. (**A**) Comparison of CmWRKY8-1 with other homologous proteins of different species. The red lines mark the WRKYGQK heptapeptide sequence as well as the zinc finger structural domain. (**B**) Phylogenetic analysis of CmWRKY8-1. Red fonts indicate CmWRKY8-1.

Previously, *CmWRKY8* (KC615362) was found to respond positively to *F. oxysporum* inoculation in chrysanthemums ‘Jinba’, suggesting that *CmWRKY8* may be involved in resistance to *F. oxysporum* in chrysanthemums [28]. We isolated a 744 bp full-length open reading frame (ORF) encoding a polypeptide of 247 amino acid residues from ‘Jinba’ (Table S1). Compared to the protein sequence of CmWRKY8, only two amino acids were found to be different. Therefore, we named the gene *CmWRKY8-1*. CmWRKY8-1 contains 27 negatively charged residues and 33 positively charged residues. The molecular weight is 27,704.47 Da, and the theoretical pI is 8.80. The instability index is computed as 48.14, which classifies the protein as unstable. The grand average hydropathicity is −1.079, which makes it a hydrophilic protein. CmWRKY8-1 contains a WRKY domain of approximately 60 amino acids that contains a WRKYGQK motif and a zinc finger motif (C-X7-C-X23-H-X1-C), belonging to class III WRKY transcription factors (Figure 1A). Phylogenetic tree analysis showed that CmWRKY8-1 had the highest sequence similarity to Artemisia annua AaWRKY8 (Figure 1B).

### 2.2. Characteristics of CmWRKY8-1

**Figure 2:**
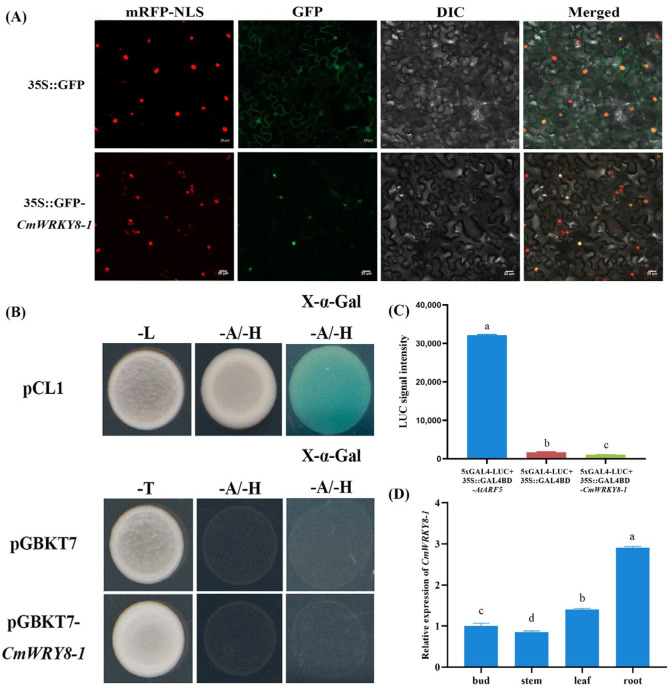
Characteristics of *CmWRKY8-1*. (**A**) Subcellular localization of CmWRKY8-1 in tobacco leaves. mRFP-NLS shows localization of 35S::D53-RFP. GFP shows localization of 35S::GFP-*CmWRKY8-1*. DIC shows epidermal cells. The merged images combine RFP with GFP and DIC signals. Bars indicate 20 μm. (**B**) Yeast validation of *CmWRKY8-1* transcriptional activation activity. The pCL1 and pGBKT7 plasmids were used as positive and negative controls, respectively. (**C**) GLOMAX chemiluminescence determination of relative luciferase activity. The different letters mean significant differences according to Duncan’s multiple range test at *p* < 0.05; the same scheme applies below. (**D**) The relative expression of *CmWRK8-1* in bud, stem, leaf and root during the nutritional growth period. The 2^−ΔΔCt^ method was used to calculate relative transcript abundances.

To determine the subcellular localization of CmWRKY8-1, the pORE R4-GFP-*CmWRKY8-1* vector or pORE R4-GFP empty vector was infiltrated into the epidermal cells of *N. benthamiana* leaves. The GFP fluorescence signal of tobacco cells transformed with the pORE R4-GFP-*CmWRKY8-1* vector appeared only in the nucleus and overlapped with the nuclear marker (Figure 2A). In conclusion, CmWRKY8-1 was localized in the nucleus.

The pDEST-GBKT7-*CmWRKY8-1* plasmid was transformed into the yeast strain Y2H to verify its transcriptional activation activity. Yeast transformed with the positive control pCL1 plasmid grew normally, whereas yeast transformed with the negative control pDEST-GBKT7 plasmid and pDEST-GBKT7-*CmWRKY8-1* plasmid did not grow normally on SD/-Ade/-His deficient medium (Figure 2B). These results indicate that *CmWRKY8-1* has no transcriptional activation activity. To further determine the transcriptional activity of *CmWRKY8-1*, we performed a transcriptional activity analysis of chrysanthemum protoplasts. The 35S::GAL4DB-*CmWRKY8-1*, 35S::GAL4DB-*AtARF5*, and 35S::GAL4DB plasmids were cotransformed with equal amounts of 5×GAL4-LUC in chrysanthemum protoplasts. *CmWRKY8-1* luciferase activity values were significantly lower than those of the positive control, as measured using the GLOMAX chemiluminescence meter (Figure 2C). Therefore, *CmWRKY8-1* showed no transcriptional activation activity.

In order to explore the expression pattern of *CmWRKY8-1* in various tissues during the vegetative period of chrysanthemums, the terminal bud, stem, leaf and root of ‘Jinba’ were sampled and analyzed by qRT-PCR. The results showed that the relative expression level was highest in the roots, followed by the leaves (Figure 2D).

### 2.3. Expression Pattern of CmWRKY8-1 after F. oxysporum Infection in C. moriflium ‘Jinba’ and Spraying Exogenous SA to Improve the Resistance of C. moriflium ‘Jinba’ to F. oxysporum

**Figure 3:**
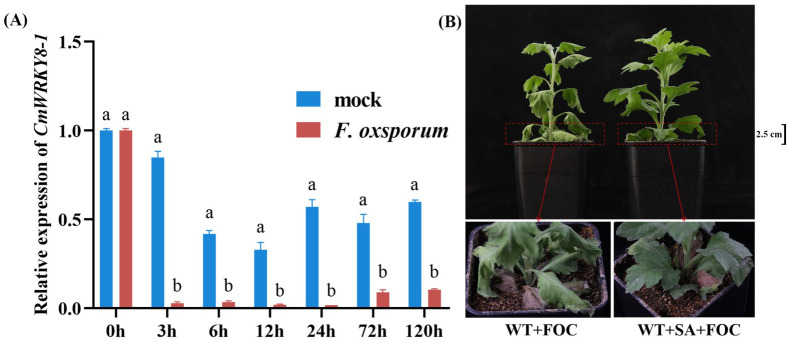
Expression of *CmWRKY8-1* under *F. oxysporum* infection and the phenotype of inoculated chrysanthemums after spraying exogenous SA. (**A**) Changes in the relative expression of *CmWRKY8-1* in ‘Jinba’ after inoculation with *F. oxysporum*. (**B**) The control group was inoculated with the fungus after 24 h of spraying sterile water and the experimental group was inoculated with the fungus after 24 h of spraying SA. FOC indicates *F. oxysporu*.

To verify whether *CmWRKY8-1* responds to *F. oxysporum* infection, we collected root samples at 0 h, 3 h, 6 h, 12 h, 24 h, 72 h, and 120 h after *F. oxysporum* inoculation in ‘Jinba’ for RNA extraction, cDNA reverse transcription, and qRT-PCR data analysis. The results showed a significant decrease in *CmWRKY8-1* expression in the experimental group compared to that in the control (Figure 3A), suggesting that *CmWRKY8-1* may play an important role in the response to *F. oxysporum*.

Recent studies have found that when *F. oxysporum* infests chrysanthemums, the salicylic acid O-β-glucoside (SAG) content in chrysanthemums increases, triggering systemic defense [29]. To investigate whether SA affected the resistance of chrysanthemums to *F. oxysporum*, we sprayed exogenous SA. The experimental group was inoculated 24 h after spraying with 200 μm SA, and the control group was inoculated 24 h after spraying with sterile water. By observation, we found that chrysanthemums sprayed with SA showed greater resistance (Figure 3B). These results indicate that SA plays a positive defensive role in chrysanthemums in response to *F. oxysporum* infestation.

### 2.4. Overexpression of CmWRKY8-1-VP64 Fusion Protein Increases the Susceptibility of Chrysanthemums to F. oxysporum

**Figure 4:**
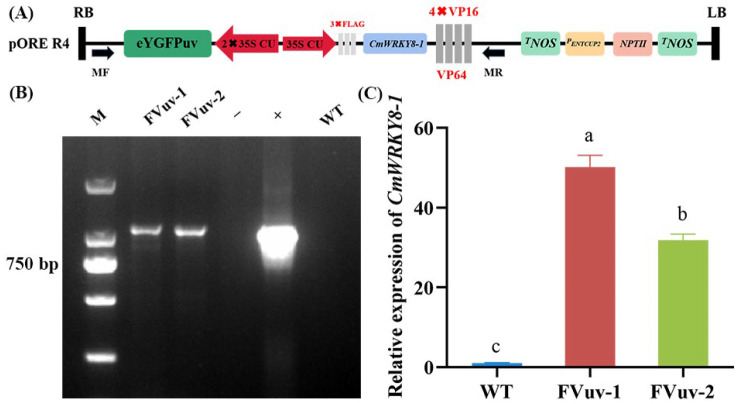
Acquisition of *CmWRKY8-1* transgenic lines. (**A**) Schematic diagram of pR4-FVuv-*CmWRKY8-1* vector. (**B**) PCR identification of transgenic lines at the DNA level using vector- and gene-specific primers. The positive control used the pR4-FVuv-*CmWRKY8-1* vector as a PCR template. The negative control used the DNA of ‘Jinba’ and water as PCR template. (**C**) Relative expression of *CmWRKY8-1* in the transgenic lines.

To clarify the function of *CmWRKY8-1*, we obtained *CmWRKY8-1* transgenic lines that overexpressed the *CmWRKY8-1–VP64* fusion protein (Figure 4A). We selected FVuv-1 and FVuv-2 as subjects for the *CmWRKY8-1* transgenic lines. Through PCR amplification with the 35S forward primer and *CmWRKY8-1* reverse primer, transgenic lines of *CmWRKY8-1* were identified (Figure 4B). The transgenic lines of *CmWRKY8-1* were further analyzed by qRT-PCR (Figure 4C).

**Figure 5:**
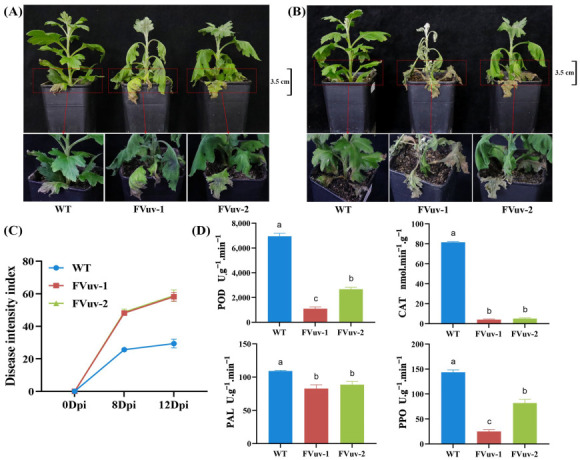
Physiological phenotypes after inoculation with *F. oxysporum*. (**A**) Phenotypes at the eighth day after inoculation with *F. oxysporum*. (**B**) Phenotypes at the twelfth day after inoculation *with F. oxysporum*. (**C**) Disease severity index of plants. (**D**) The content of POD, CAT, PAL, and PPO on the 8th day of inoculation.

After inoculation with *F. oxysporum*, we observed that FVuv-1 and FVuv-2 already showed a clear wilting state on the 8th day, whereas WT lines had only a slight wilting at the base (Figure 5A). On the 12th day, the transgenic seedling disease of the FVuv-1 and FVuv-2 lines had spread from the middle to the top of the stem, and the entire plant had died (Figure 5B). The WT lines still had only a few leaves withered at the base. We evaluated and graded chrysanthemum seedlings manually for the degree of root browning and the degree of yellowing and browning of leaves in diseased chrysanthemum seedlings. The DSI of WT lines was significantly lower than that of FVuv-1 and FVuv-2 (Figure 5C). More importantly, we measured the activities of peroxidase (POD), catalase (CAT), phenylalaninammo-nialyase (PAL), and polyphenol oxidase (PPO) enzymes in plants after infection on the 8th day. POD, CAT, PAL, and PPO are critical enzymes in plant defense systems, and can be used as standards to measure plant resistance [30,31]. The results showed that enzyme contents in FVuv-1 and FVuv-2 were significantly lower than those in WT lines (Figure 5D).

These results suggest that the overexpression of *CmWRKY8-1*-VP64 fusion protein can improve the susceptibility of chrysanthemums to *F. oxysporum*.

### 2.5. Changes in Genes Involved in the SA Signaling Pathway and Alterations in Endogenous SA

**Figure 6:**
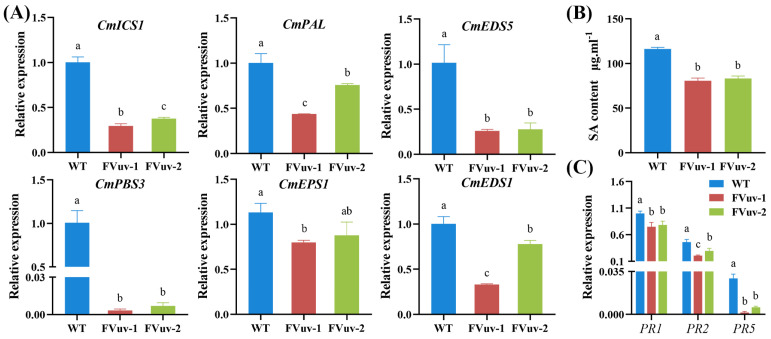
Changes in genes involved in SA signaling pathway and alterations in endogenous SA. (**A**) Expression of *CmICS1*, *CmPAL*, *CmEDS5*, *CmPBS3*, *CmEPS1*, *CmEDS1* in *CmWRKY8-1* transgenic lines and WT after inoculation with *F. oxysporum*. (**B**) Endogenous SA content after inoculation with *F. oxysporum*. (**C**) Expression of pathogenesis-related genes involved in the SA signaling pathway.

Plants can synthesize SA via ICS and PAL pathways. *EDS5*, *PBS3*, *EDS1*, and *EPS1* are involved in the SA pathway [32]. To investigate whether *CmWRKY8-1* is involved in regulating the response to *F. oxysporum* through SA, we determined the genes related to the SA pathway using qRT-PCR after inoculation with *F. oxysporum*. The results showed that the relative expression levels of *ICS1*, *PAL*, *EDS5*, *PBS3*, *EPS1*, and *EDS1* in *CmWRKY8-1* transgenic lines were significantly lower than those in WT lines (Figure 6A). We also determined the endogenous SA content of plants after inoculation with *F. oxysporum*. We observed that the content of endogenous SA in *CmWRKY8-1* transgenic lines was significantly lower than that in the WT lines (Figure 6B). Therefore, *CmWRKY8-1* may participate in the SA pathway in response to *F. osporium* infection. To determine whether *CmWRKY8-1* could directly respond to *F. oxysporum* infection through the SA pathway, we measured the expression of disease-resistant defense genes downstream of SA. Here, we measured the relative expression of *PR1*, *PR2*, and *PR5* after inoculation with *F. oxysporum* by qRT-PCR. The results showed that the transcription levels of *PR1*, *PR2*, and *PR5* in *CmWRKY8-1* transgenic lines were generally lower than those in the WT lines (Figure 6C).

Therefore, we concluded that *CmWRKY8-1* transgenic lines could potentially reduce endogenous SA by inhibiting the expression of SA-related genes, which decreases the expression of downstream disease-related proteins, leading to susceptibility to disease.

### 2.6. Transcriptome Sequencing Analysis and Functional Enrichment of DEGs in CmWRKY8-1 Transgenic Lines

We performed transcriptome sequencing to better elucidate the expression profiles of WT lines and *CmWRKY8-1* transgenic chrysanthemum lines. The roots of WT lines and *CmWRKY8-1* transgenic lines at 0 h, 3 h, and 72 h after infection were sampled with three biological replicates, and total RNA was extracted for sequencing. In total, 861,033,134 reads were generated, resulting in 856,387,816 clean reads after filtration. 

**Figure 7:**
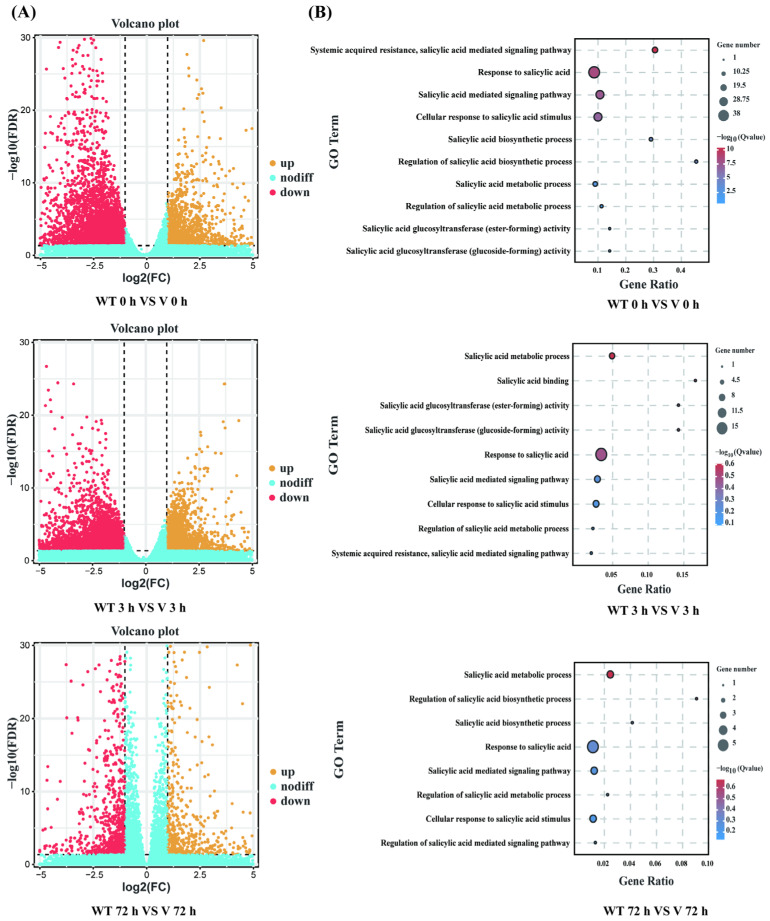
Global analysis of the transcriptome data and DEGs analysis of WT and *CmWRKY8-1* transgenic lines. (**A**) Up- or down-regulated differential gene volcano map between WT and *CmWRKY8-1* transgenic lines. (**B**) Go terms related to the SA pathway.

**Figure 8:**
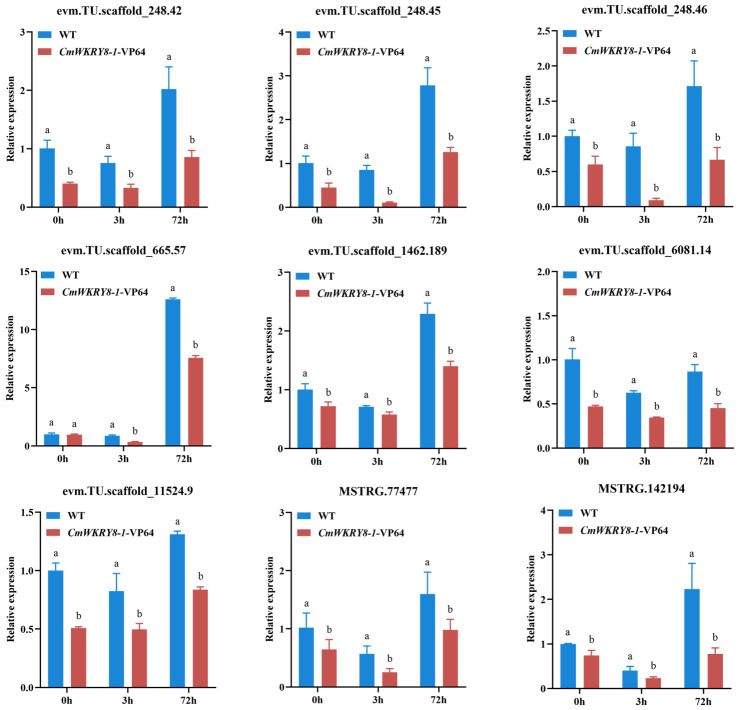
The expression levels of DEGs to the salicylate signaling pathway between WT and *CmWRKY8-1* transgenic lines at 0 h, 3 h, and 72 h after inoculation treatment.

Biological replicates in different groups showed a high degree of consistency, while both WT lines and *CmWRKY8-1* transgenic lines in 72 h showed different expression profiles compared to the other groups (Figure S1). Differentially expressed genes (DEGs) were identified using the following threshold criteria: FDR ≤ 0.05 and |log2| ≥ 1. There were 5745, 5840, and 1527 DEGs between the WT and *CmWRKY8-1* transgenic lines at 0 h, 3 h, and 72 h, respectively (Figure 7A). The Venn diagram showed that there were 191 DEGs in the three comparisons (Figure S2). To investigate the function of differential genes at each time point, the DEGs of three time-point comparisons between WT lines and transgenic lines were annotated using GO analysis (Figure 7B). In the GO enrichment analysis, SA-associated pathways were enriched, including “Response to salicylic acid,” “Salicylic acid mediated signaling pathway,” “Salicylic acid metabolic process,” “Cellular response to salicylic acid stimulus,” “Systemic acquired resistance”, and “salicylic acid mediated signaling pathway”. To further verify the function of SA, we quantified the expression of genes in the SA pathway (Figure S3). Compared with WT plants, the expression levels of two transcripts of *PAL* (evm.TU.scaffold_665.57, evm.TU.scaffold_1462.189), four transcripts of *EDS1* (evm.TU.scaffold_6081.14, evm.TU.scaffold_248.42, evm.TU.scaffold_248.45, evm.TU.scaffold_248.46), one transcript of *NPR1* (evm.TU.scaffold_11524.9), and two transcripts of *AIM1* (MSTRG.77477, MSTRG.142194) were lower in *CmWRKY8-1* transgenic lines. To verify the authenticity of the transcriptome data, we validated the DEGs using qRT-PCR against the SA pathway described above. The results showed that the trends of transcript expression changes obtained by qRT-PCR were consistent with the transcriptomic data (Figure 8). This indicated that *CmWRKY8-1* could influence *F. oxysporum* infection by mediating the SA pathway genes.

## 3. Discussion

In this experiment, we demonstrated that the *CmWRKY8-1* transgenic strain increased susceptibility to *F. oxysproum* by overexpressing *CmWRKY8-1*-VP64. Furthermore, we found that *CmWRKY8-1* could influence the resistance of ‘Jinba’ to *F. oxysproum* by regulating the SA pathway through transcriptomic data analysis. WRKY transcription factors play a complex regulatory role in plant defence signaling pathways. Currently, an increasing number of studies on WRKY responses to *F. oxysporum* infestation are also being conducted. *LrWRKY3* heterologously specializes in tobacco, which exhibits enhanced resistance to *F. oxysporum* infestation, and transient expression of the *LrWRKY3* RNAi vector in *Lilium regale* scales increased the susceptibility to *F. oxysporum* [33]. In cotton, group IIc WRKY TFs enhance resistance to *F. oxysporum* by promoting flavonoid synthesis via the WRKY-MAPK pathway [34]. In chickpeas, *CaWRKY70* inhibits multiple immune signaling pathways, including CaMPK9-CaWRKY40 signaling, thereby negatively regulating resistance to *F. oxysporum* [17,35]. However, there are few reports on WRKY-responsive *F. oxysporum* in chrysanthemums. To investigate the function of WRKY transcription factors in response to *F. oxysporum* infestation, we selected and cloned *CmWRKY8-1* based on previous results [28]. *CmWRKY8-1* is a transcription factor that is localized in the nucleus of cells (Figure 2A,B). Based on quantitative tissue analysis, *CmWRKY8-1* was found to be highly expressed in roots (Figure 2D). We speculate that the high expression of *CmWRKY8-1* in the roots may facilitate its positive response to *F. oxysporum* infestation.

In the study of transcription factors, the fusion of transcription factors with VP64 proteins can have a more significant regulatory effect if VP64 can convert transcriptional repressors into transcriptional activators [36]. We took advantage of the characteristics of VP64 and fused CmWKRY8-1, which has no transcriptional activation activity, to VP64 to make it a transcriptional activator. Transgenic chrysanthemum lines overexpressing CmWKRY8-1-VP64 were generated using transgenic techniques. *CmWKRY8-1* transgenic lines exhibited reduced resistance to *F. oxysporum* (Figure 5).

Plants have intricate defense systems and plant hormones play a vital role. SA plays an important role in plant immune processes by inducing systemic acquired resistance (SAR) in plants [37,38]. The synthesis of SA in plants is divided into PAL and ICS pathways, and it has been shown that the ICS pathway produces most of the SA produced by disease resistance induction [39,40]. SA biosynthesis and signal transduction are enhanced in plants upon pathogen infestation, and SA induces the expression of disease resistance-related genes, thereby improving plant resistance to disease. When *F. oxysporum* infested chrysanthemums, the SAG content in chrysanthemums increased [29]. Our experiments also demonstrated that chrysanthemum resistance to *F. oxysporum* was enhanced when exogenous SA was sprayed (Figure 3B). In soybean, *GmWRKY31* mediates resistance to *Peronospora manshurica* by regulating the expression of the *GmSAGT1* gene and participating in the SA pathway [41]. In a study on cotton, the GhMKK4–GhMPK20–GhWRKY40 cascade was found to reduce resistance to *F. oxysporum* by blocking the SA-mediated defense pathway [42]. Silencing *GhWRKY70* in cotton can reduce the sensitivity of cotton to *Verticillium dahliae* by downregulating SA gene expression [43]. In woody plants, the SA-mediated defense genes *PR1*, *PR2*, and *PAD4* were significantly upregulated in poplar after the overexpression of *PtrWRKY73* in vivo [44]. It has also been shown that *CmWRKY15-1* can enhance resistance to *Puccinia horiana Henn* through the SA pathway by interacting with *NPR1* [45]. In the present study, the key genes of the SA synthesis pathway were detected by qRT-PCR. We observed that the relative expression of all these genes was lower in *CmWRKY8-1* transgenic lines than in WT lines after inoculation (Figure 6A). In the transgenic *CmWRKY8-1* lines, the endogenous SA content was lower than that of the WT lines (Figure 6B). Therefore, we tentatively hypothesized that *CmWRKY8-1* might be involved in the SA signaling pathway and may affect the synthesis and degradation of SA. We also focused on PR genes downstream of SA. The relative expression levels of *PR1*, *PR2*, and *PR5* were lower in the *CmWRKY8-1* transgenic lines than in the WT lines (Figure 6C). RNA-seq analysis revealed that the relative expression of DEGs associated with the SA pathway was lower in *CmWRKY8-1* transgenic lines (Figure 8). A GO analysis revealed that GOs were associated with the SA signaling pathway (Figure 7B). In summary, we suggest that *CmWRKY8-1* affects resistance to *F. oxysporum* by regulating the SA pathway. However, the mechanism of how *CmWRKY8-1* directly or indirectly regulates the SA pathway needs to be further explored.

Under the conditions of high temperature, high humidity and continuous cultivation, large amounts of *F. oxysporum* will accumulate in the soil, resulting in the frequent occurrence of chrysanthemum Fusarium wilt. We expect to apply transgenic chrysanthemum to field cultivation, screen the resistant germplasm of chrysanthemum by transgenic means, and cultivate resistant and high-quality varieties. We also hope to link the environment, molecule and *F. oxysporum* to study the disease resistance mechanism of chrysanthemum, improving the quality of chrysanthemum from many angles.

## 4. Materials and Methods

### 4.1. Plant Materials and Growth Conditions

The chrysanthemum variety ‘Jinba’ used in this experiment was provided by the Chrysanthemum Germplasm Resource Preserving Center, Nanjing Agricultural University (Nanjing, China). Chrysanthemum cuttings were planted in a 1:2 (*v*/*v*) mixture of soil and vermiculite. Chrysanthemums were grown in a greenhouse with a photoperiod of 16 h/8 h (light/dark), a temperature of 25 °C, and a humidity of 70%.

### 4.2. Isolation, Identification, Culture and Inoculation of Pathogenic Fungus

The diseased parts of plants were observed in the field, and the diseased plants were collected. The fungus was isolated by the tissue block method and inoculated on a PDA plate after purification. The colony morphology was observed, and the genomic DNA of fungus was extracted and sequenced. Finally, the fungal species was observed by re-inoculation and phenotyping.

*F. oxysporum* used in the experiment was isolated from the root and soil of chrysanthemum variety ‘Jinba’ at the Chrysanthemum Germplasm Resource Preserving Center (Nanjing, China) and stored at −80 °C. Before inoculation, the preserved fungal cakes were inoculated on PDA plates and cultured at 28 °C for six days. Then, 10 fungal cakes with a diameter of 0.7 cm were cut and inoculated into 500 mL PDB medium at 28 °C and 180 rpm, and the culture was shaken for 5 days. Spore concentration was determined using a blood counting plate. For inoculation, chrysanthemum roots were cut lightly and soaked in a spore suspension at a concentration of 10^7^ CFU ml^−1^ for 30 min. When each plant finally colonized, the seedlings were inoculated with 1 × 10^7^ spores per gram of substrate. The chrysanthemums were cultured in an environment of 16 h/8 h (light/dark), 28 °C and 80% humidity in a photo-culture chamber.

### 4.3. Isolation and Sequence Analysis of CmWRKY8-1

Total RNA was extracted from snap-frozen roots of the cultivar ‘Jinba’ using an RNA extraction kit (HuaYueyang), and reverse transcription was performed using a rapid reverse transcription kit (TaKaRa) to obtain cDNA as a template for gene cloning. Primers (Table S2) were designed for PCR amplification according to the gene sequences logged in the NCBI database (KC615362). PCR products were purified, constructed into the pMD19-T (TaKaRa) vector, and sequenced. The CmWRKY8-1 homologue peptide sequence was retrieved from TAIR (https://www.arabidopsis.org/, accessed on 10 October 2022) and the NCBI web site (https://www.ncbi.nlm.nih.gov, accessed on 10 October 2022). CmWRKY8-1 was used to perform multiple sequence alignments of homologous sequences using DNAMAN6, and MEGA X was used to construct phylogenetic trees using the adjacency method. The online tool ExPASy (http://expasy.org, accessed on 10 October 2022) was used to predict the physicochemical properties of CmWRKY8-1.

### 4.4. Subcellular Localization of CmWRKY8-1

The ORF of *CmWRKY8-1* was constructed using the pORE-R4 (35S::GFP) vector. pORE-R4-*CmWRKY8-1* and 35S::D53-RFP were transferred to the *Agrobacterium tumefaciens* strain GV3101. They were then transiently cotransformed into *Nicotiana benthamiana* leaves [46], and 35S::D53-RFP was used as a nuclear marker. The co-transformed leaves were observed using a Zeiss LSM 780 confocal microscope (Zeiss, Jena, Germany).

### 4.5. Transactivation Assays of CmWRKY8-1

A yeast assay was performed to examine the transcriptional activity of *CmWRKY8-1* [47]. The pGBKT7-*CmWRKY8-1* vector was constructed. pCL1 (positive control), pGBKT7 (negative control), and pGBKT7-*CmWRKY8-1* were transformed into the yeast cells Y2HGold. pCL1 was coated in SD/Leu-media, and the pGBKT7 and pGBKT7-*CmWRKY8-1* vectors were coated in SD/Trp-media and incubated for 3–5 days at 28 °C in an inverted position. Finally, colonies were picked and spotted according to different concentration gradients plates onto SD/Ade-His-media with and without X-α-gal, and incubated overnight at 28 °C in an inverted position. Colonies were observed and photographed.

The detection of the transcriptional activation activity of *CmWRKY8-1* was conducted by the chrysanthemum protoplast system. High-concentration plasmids 35S::GAL4DB-*CmWRKY8-1*, 35S::GAL4DB-*AtARF5*, 35S::GAL4DB, and 5×GAL4-LUC plasmids were prepared. Chrysanthemum protoplasts were extracted from the leaves of young one-month-old chrysanthemum histoculture seedlings by referring to the transformation method of *Arabidopsis protoplasts* [48]. 35S::GAL4DB-*CmWRKY8-1* and 5×GAL4-LUC were cotransformed into chrysanthemum protoplasts to determine whether *CmWRKY8-1* had transcriptional activation or repression activity. The positive control group was 35S::GAL4DB-*AtARF5*+5×GAL4-LUC. 35S::GAL4DB empty vector+5×GAL4-LUC in the negative control group and 35S::GAL4DB-*CmWRKY8-1*+5×GAL4-LUC in the experimental group. LUC luminescence were measured using a GLOMAX^®^-20/20 instrument.

### 4.6. Quantitative Real-Time PCR (qRT-PCR)

Quantitative primers (Table S2) were designed using Primer5, and EF1α (Table S2) was used as the internal reference gene [49]. qRT-PCR was performed using the SYBR Green PCR Master Mix (TaKaRa). The 96-well plate was placed in a Mastercycler ep realplex 2S (Eppendorf, Hamburg, Germany) qRT-PCR instrument, and the fluorescence acquisition channel and fluorescence reading step were set. PCR reactions were performed according to the following reaction conditions: 95 °C for 2 min, 95 °C 15 s, 55 °C 15 s, 72 °C 20 s, 40 cycles. Finally, the dissolution curve program was developed. Calculations were performed using the 2^−ΔΔCt^ method [50].

### 4.7. Analysis of CmWRKY8-1 Expression under Stress Treatments

40 days old seedlings were used to determine the expression patterns of *CmWRKY8-1* under different stress treatments. For the inoculation treatment, the experimental group was treated with *F. oxysporum*, whereas the control group was treated with sterile water. Root samples were collected at 0 h, 3 h, 6 h, 12 h, 24 h, 72 h, and 120 h after treatment with *F. oxysporum*. For the SA treatment, the experimental group was sprayed with 200 μm SA, while the control group was sprayed with sterile water.

### 4.8. Generation of CmWRKY8-1 Transgenic Chrysanthemum

*CmWRKY8-1* was fused to the VP64 (4 × VP16) protein to produce the transcriptional activator R4-FVuv-*CmWRKY8-1*. The vector was introduced into *Agrobacterium tumefaciens* strain EHA105 and ‘Jinba’ was transformed by Agrobacterium-mediated transformation [51]. Transgenic chrysanthemums were primed with vector primers and *CmWRKY8-1* specific primers (Table S2) for DNA-level identification. qRT-PCR analysis at the RNA level was performed using *CmWRKY8-1* quantitative primers.

### 4.9. Analysis of F. oxysporum Resistance in CmWRKY8-1 Transgenic Chrysanthemums

Twenty seedlings of 40 days old wild-type and transgenic chrysanthemums were used for inoculation treatment. The disease severity index (DSI) was calculated on days 8 and 12 after inoculation, and the phenotypes were photographed and recorded. The DSI was calculated according to the following formula: DSI = ∑ (number of diseased plants per level × number of corresponding levels) ×100 / (total number of plants surveyed × highest disease level) (Table S3).

### 4.10. Enzyme Activity Assay and Endogenous SA Determination

Root samples were taken from transgenic lines and WT lines on the 8th day after inoculation. The enzyme activity in chrysanthemums was measured using POD, CAT, PAL, and PPO kits (Comin, Suzhou, China). In the meantime, the endogenous SA content was determined by a Plant Hormone Salicylic Acid ELISA Kit (Lengton Bioscience Co., Shanghai, China).

### 4.11. RNA-Seq Analysis

Seedlings of the WT lines and *CmWKRY8-1* transgenic lines were planted in a greenhouse at Nanjing Agricultural University for 40 days. After inoculation at 0 h, 3 h, and 72 h, roots of WT lines and *CmWKRY8-1* transgenic lines were sampled with three biological replicates. Total RNA was extracted using a TRIzol reagent kit (Invitrogen, Carlsbad, CA, USA) and assessed using an Agilent 2100 Bioanalyzer (Agilent Technologies, Palo Alto, CA, USA). Transcriptome sequencing was performed using an Illumina Novaseq6000 made by the Gene Denovo Biotechnology Co. (Guangzhou, China). The reads were filtered and aligned using Bowtie2 (version 2.2.8) [52]. Paired-end clean reads were mapped to the reference genome using the HISAT2 software version 2.4 [53]. Differentially expressed genes (DEGs) between two different groups were identified using DESeq2 software [54]. The transcripts were annotated using the BLASTX algorithm [55] and Gene Ontology database [56]. The quantification of transcripts was transformed into FPKM values with RSEM [57].

### 4.12. Data Analysis

Statistical analyses were performed using SPSS version 25.0. All data were analyzed using analysis of variance (ANOVA) and *t*-tests to determine significant differences.

## 5. Conclusions

In conclusion, we identified a WRKY gene in chrysanthemums, *CmWRKY8-1*, and found that it was responsive to *F. oxysporum* infestation. By overexpressing *CmWRKY8-1*-VP64, we found that the resistance of chrysanthemums to *F. oxysporum* was reduced. Moreover, the expression of genes related to the SA signaling pathway and endogenous SA content was decreased in *CmWRKY8-1* transgenic lines. RNA-seq analysis showed that the expression of DEGs of the SA signaling pathway was decreased in *CmWRKY8-1* transgenic lines. Therefore, we demonstrated that *CmWRKY8-1* responds to *F. oxysporum* infection by regulating the SA signaling pathway.

## Data Availability

The datasets presented in this study can be found in online repositories. The names of the repository/repositories and accession number(s) can be found below: National Center for Biotechnology Information (NCBI) BioProject database under accession number PRJNA896484.

## Supplementary Materials

The following supporting information can be downloaded at: https://www.mdpi.com/article/10.3390/ijms24043499/s1.

## References

[1] Ploetz R.C. (2006). Fusarium wilt of banana is caused by several pathogens referred to as *Fusarium oxysporum *f. sp.* cubense*. Phytopathology.

[2] Minuto A., Gullino M.L., Garibaldi A. (2007). *Gerbera jamesonii, Osteospermum* sp. and *Argyranthemum frutescens*: New hosts of *Fusarium oxysporum* f. sp. chrysanthemi. J. Phytopathol..

[3] Chen X., Shu Y., Luo M., Xiang M., Huang Y., Zhang W., Dong Z. (2020). Fusarium wilt of imperial Chrysanthemum (*Chrysanthemum morifolium*) caused by *Fusarium oxysporum* in China. Plant Dis..

[4] DeFalco T.A., Zipfel C. (2021). Molecular mechanisms of early plant pattern-triggered immune signaling. Mol. Cell.

[5] Zhai K., Di Liang D., Li H., Jiao F., Yan B., Liu J., Lei Z., Huang L., Gong X., Wang X. (2022). NLRs guard metabolism to coordinate pattern- and effector-triggered immunity. Nature.

[6] Rushton P.J., Somssich I.E., Ringler P., Shen Q.J. (2010). WRKY transcription factors. Trends Plant Sci..

[7] Wani S.H., Anand S., Singh B., Bohra A., Joshi R. (2021). WRKY transcription factors and plant defense responses: Latest discoveries and future prospects. Plant Cell Rep..

[8] Zhang Y., Wang L. (2005). The WRKY transcription factor superfamily: Its origin in eukaryotes and expansion in plants. BMC Evol. Biol..

[9] Rinerson C.I., Rabara R.C., Tripathi P., Shen Q.J., Rushton P.J. (2015). The evolution of WRKY transcription factors. BMC Plant Biol..

[10] Song H., Sun W., Yang G., Sun J. (2018). WRKY transcription factors in legumes. BMC Plant Biol..

[11] Ülker B., Somssich I.E. (2004). WRKY transcription factors: From DNA binding towards biological function. Curr. Opin. Plant Biol..

[12] Jiang J., Ma S., Ye N., Jiang M., Cao J., Zhang J. (2017). WRKY transcription factors in plant responses to stresses. J. Integr. Plant Biol..

[13] Chen L., Zhang L., Yu D. (2010). Wounding-induced wrky8 is involved in basal defense in *Arabidopsis*. Mol. Plant-Microbe Interact..

[14] Zheng Z., Abu Qamar S., Chen Z., Mengiste T. (2006). Arabidopsis WRKY33 transcription factor is required for resistance to necrotrophic fungal pathogens. Plant J..

[15] Jiang Y., Yu D. (2016). The WRKY57 transcription factor affects the expression of Jasmonate ZIM-domain genes transcriptionally to compromise *Botrytis cinerea* resistance. Plant Physiol..

[16] Wang W.J., Li T., Chen Q., Yao S.X., Deng L.L., Zeng K.F. (2022). CsWRKY25 improves resistance of citrus fruit to *Penicillium digitatum* via modulating reactive oxygen species production. Front Plant Sci..

[17] Chakraborty J., Ghosh P., Sen S., Nandi A.K., Das S. (2019). CaMPK9 increases the stability of CaWRKY40 transcription factor which triggers defense response in chickpea upon *Fusarium oxysporum f.* sp. *ciceri* Race1 infection. Plant Mol. Biol..

[18] Zhang W., Gao T., Li P., Tian C., Song A., Jiang J., Guan Z., Fang W., Chen F., Chen S. (2020). Chrysanthemum CmWRKY53 negatively regulates the resistance of chrysanthemum to the aphid *Macrosiphoniella sanborni*. Hortic. Res..

[19] Vlot A.C., Dempsey D.M.A., Klessig D.F. (2009). Salicylic acid, a multifaceted hormone to combat disease. Annu. Rev. Phytopathol..

[20] Bi M., Li X., Yan X., Liu D., Gao G., Zhu P., Mao H. (2021). Chrysanthemum WRKY15-1 promotes resistance to *Puccinia horiana Henn*. via the salicylic acid signaling pathway. Hortic. Res..

[21] Zhao X., Qi C., Jiang H., Zhong M., You C., Li Y., Hao Y. (2020). MdWRKY15 improves resistance of apple to *Botryosphaeria dothidea* via the salicylic acid-mediated pathway by directly binding the *MdICS1* promoter. J. Integr. Plant Biol..

[22] Yaghmai R., Cutting G.R. (2002). Optimized regulation of gene expression using artificial transcription factors. Mol. Ther..

[23] Lowder L.G., Zhang D., Baltes N.J., Paul J.W., Tang X., Zheng X., Voytas D.F., Hsieh T.-F., Zhang Y., Qi Y. (2015). A CRISPR/Cas9 Toolbox for multiplexed plant genome editing and transcriptional regulation. Plant Physiol..

[24] Piatek A., Ali Z., Baazim H., Li L., Abulfaraj A., Al-Shareef S., Aouida M., Mahfouz M.M. (2015). RNA-guided transcriptional regulation *in planta* via synthetic dCas9-based transcription factors. Plant Biotechnol. J..

[25] Ren C., Li H., Liu Y., Li S., Liang Z. (2022). Highly efficient activation of endogenous gene in grape using CRISPR/dCas9-based transcriptional activators. Hortic. Res..

[26] Wang J., Wu F., Zhu S., Xu Y., Cheng Z., Wang J., Li C., Sheng P., Zhang H., Cai M. (2016). Overexpression of OsMYB1R1-VP64 fusion protein increases grain yield in rice by delaying flowering time. FEBS Lett..

[27] Hadizadeh H., Samiei L., Shakeri A. (2022). Chrysanthemum, an ornamental genus with considerable medicinal value: A comprehensive review. S. Afr. J. Bot..

[28] Song A., Li P., Jiang J., Chen S., Li H., Zeng J., Shao Y., Zhu L., Zhang Z., Chen F. (2014). Phylogenetic and transcription analysis of chrysanthemum WRKY transcription Factors. Int. J. Mol. Sci..

[29] Guan Y., He X., Wen D., Chen S., Chen F., Chen F., Jiang Y. (2022). *Fusarium oxysporum* infection on root elicit aboveground terpene production and salicylic acid accumulation in *Chrysanthemum morifolium*. Plant Physiol. Biochem..

[30] Janská A., Maršík P., Zelenková S., Ovesná J. (2010). Cold stress and acclimation—What is important for metabolic adjustment?. Plant Biol..

[31] Saruhan N., Saglam N.S., Kadioglu A. (2012). Salicylic acid pretreatment induces drought tolerance and delays leaf rolling by inducing antioxidant systems in maize genotypes. Acta Physiol. Plant..

[32] Janda T., Szalai G., Pál M. (2020). Salicylic acid signalling in plants. Int. J. Mol. Sci..

[33] Wang Z., Deng J., Liang T., Su L., Zheng L., Chen H., Liu D. (2022). Lilium regale Wilson WRKY3 modulates an antimicrobial peptide gene, *LrDef1*, during response to *Fusarium oxysporum*. BMC Plant Biol..

[34] Wang L., Guo D., Zhao G., Wang J., Zhang S., Wang C., Guo X. (2022). Group IIc WRKY transcription factors regulate cotton resistance to *Fusarium oxysporum* by promoting GhMKK2 -mediated flavonoid biosynthesis. New Phytol..

[35] Chakraborty J., Sen S., Ghosh P., Jain A., Das S. (2020). Inhibition of multiple defense responsive pathways by CaWRKY70 transcription factor promotes susceptibility in chickpea under *Fusarium oxysporum* stress condition. BMC Plant Biol..

[36] Konermann S., Brigham M.D., Trevino A.E., Joung J., Abudayyeh O.O., Barcena C., Hsu P.D., Habib N., Gootenberg J.S., Nishimasu H. (2015). Genome-scale transcriptional activation by an engineered CRISPR-Cas9 complex. Nature.

[37] Cao H., Bowling S.A., Gordon A.S., Dong X. (1994). Characterization of an Arabidopsis mutant that is nonresponsive to inducers of systemic acquired resistance. Plant Cell.

[38] Cao H., Glazebrook J., Clarke J.D., Volko S., Dong X. (1997). The arabidopsis NPR1 gene that controls systemic acquired resistance encodes a novel protein containing ankyrin repeats. Cell.

[39] Wildermuth M.C., Dewdney J., Wu G., Ausubel F.M. (2001). Erratum: Corrigendum: Isochorismate synthase is required to synthesize salicylic acid for plant defence. Nature.

[40] Gawroński P., Górecka M., Bederska M., Rusaczonek A., Ślesak I., Kruk J., Karpiński S. (2013). Isochorismate synthase 1 is required for thylakoid organization, optimal plastoquinone redox status, and state transitions in *Arabidopsis thaliana*. J. Exp. Bot..

[41] Dong H., Tan J., Li M., Yu Y., Jia S., Zhang C., Wu Y., Liu Y. (2019). Transcriptome analysis of soybean WRKY TFs in response to *Peronospora manshurica* infection. Genomics.

[42] Wang C., He X., Li Y., Wang L., Guo X., Guo X. (2018). The cotton MAPK kinase GhMPK20 negatively regulates resistance to *Fusarium oxysporum* by mediating the MKK4-MPK20-WRKY40 cascade. Mol. Plant Pathol..

[43] Xiong X., Sun S., Li Y., Zhang X., Sun J., Xue F. (2019). The cotton WRKY transcription factor GhWRKY70 negatively regulates the defense response against *Verticillium dahliae*. Crop. J..

[44] Duan Y., Jiang Y., Ye S., Karim A., Ling Z., He Y., Yang S., Luo K. (2015). PtrWRKY73, a salicylic acid-inducible poplar WRKY transcription factor, is involved in disease resistance in *Arabidopsis thaliana*. Plant Cell Rep..

[45] Gao G., Jin R., Liu D., Zhang X., Sun X., Zhu P., Mao H. (2022). CmWRKY15-1 promotes resistance to Chrysanthemum white rust by regulating CmNPR1 expression. Front. Plant Sci..

[46] Cheng P.L., Liu Y.N., Yang Y.M., Chen H., Cheng H., Hu Q., Zhang Z.X., Gao J.J., Zhang J.X., Ding L. (2020). *CmBES1* is a regulator of boundary formation in chrysanthemum ray florets. Hortic. Res..

[47] Gao H., Song A., Zhu X., Chen F., Jiang J., Chen Y., Sun Y., Shan H., Gu C., Li P. (2012). The heterologous expression in Arabidopsis of a chrysanthemum Cys2/His2 zinc finger protein gene confers salinity and drought tolerance. Planta.

[48] Yoo S.D., Cho Y.H., Sheen J. (2007). Arabidopsis mesophyll protoplasts: A versatile cell system for transient gene expression analysis. Nat. Protoc..

[49] Gu C., Chen S., Liu Z., Shan H., Luo H., Guan Z., Chen F. (2011). Reference gene selection for quantitative real-time PCR in Chrysanthemum subjected to biotic and abiotic stress. Mol. Biotechnol..

[50] Pfaffl M.W. (2001). A new mathematical model for relative quantification in real-time RT-PCR. Nucleic Acids Res..

[51] Zhu L., Guan Y., Zhang Z., Song A., Chen S., Jiang J., Chen F. (2020). CmMYB8 encodes an R2R3 MYB transcription factor which represses lignin and flavonoid synthesis in chrysanthemum. Plant Physiol. Biochem..

[52] Langmead B., Salzberg S.L. (2012). Fast gapped-read alignment with Bowtie 2. Nat. Methods.

[53] Kim D., Langmead B., Salzberg S.L. (2015). HISAT: A fast spliced aligner with low memory requirements. Nat. Methods.

[54] Love M.I., Huber W., Anders S. (2014). Moderated estimation of fold change and dispersion for RNA-seq data with DESeq2. Genome Biol..

[55] Altschul S.F., Madden T.L., Schäffer A.A., Zhang J., Zhang Z., Miller W., Lipman D.J. (1997). Gapped BLAST and PSI-BLAST: A new generation of protein database search programs. Nucleic Acids Res..

[56] Gene Ontology Consortium (2004). The Gene Ontology (GO) database and informatics resource. Nucleic Acids Res..

[57] Li B., Dewey C.N. (2011). RSEM: Accurate transcript quantification from RNA-Seq data with or without a reference genome. BMC Bioinform..

