# Supplementary material for: Overexpression of CmWRKY8-1–VP64 Fusion Protein Reduces Resistance in Response to Fusarium oxysporum by Modulating the Salicylic Acid Signaling Pathway in Chrysanthemum morifolium
Source: Int J Mol Sci. 2023 Feb 9;24(4):3499. doi: 10.3390/ijms24043499 (PMC9964100; doi:10.3390/ijms24043499)
Supplement: Supplementary file 1 [file ijms-24-03499-s001.zip › Supplement of figures.pdf]

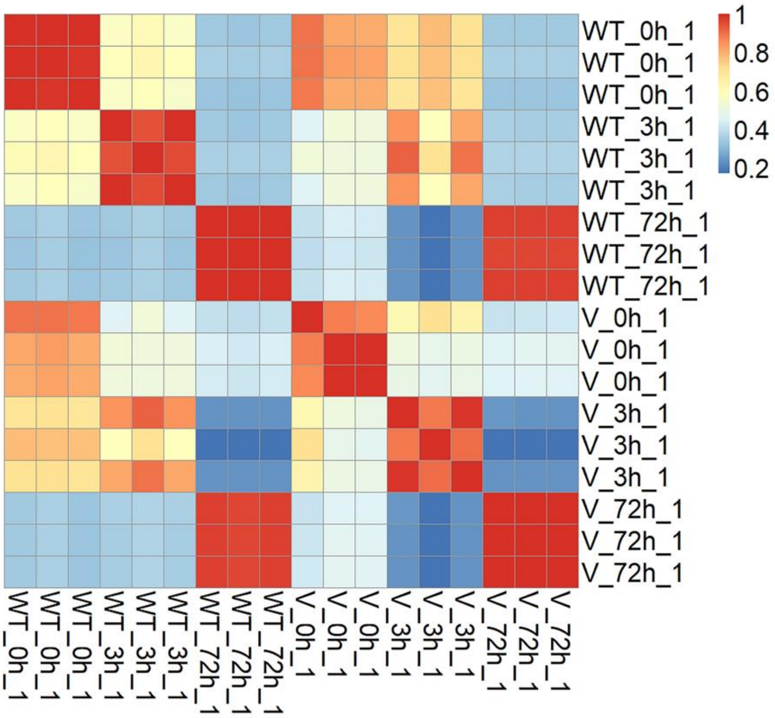

FIGURE S1| Pearson's correlation between eighteen samples.

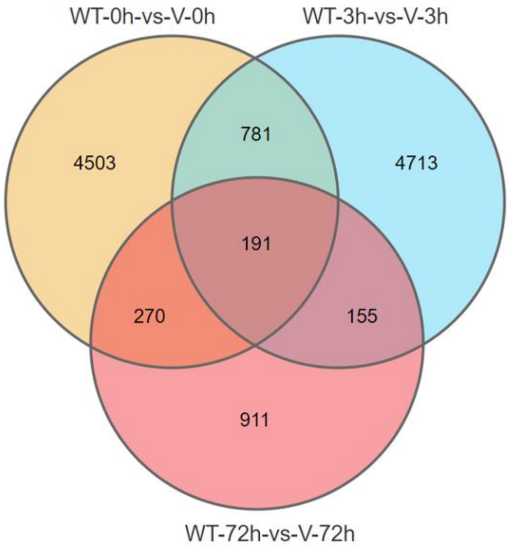

FIGURE S2| The Venn diagram in the three comparisons.

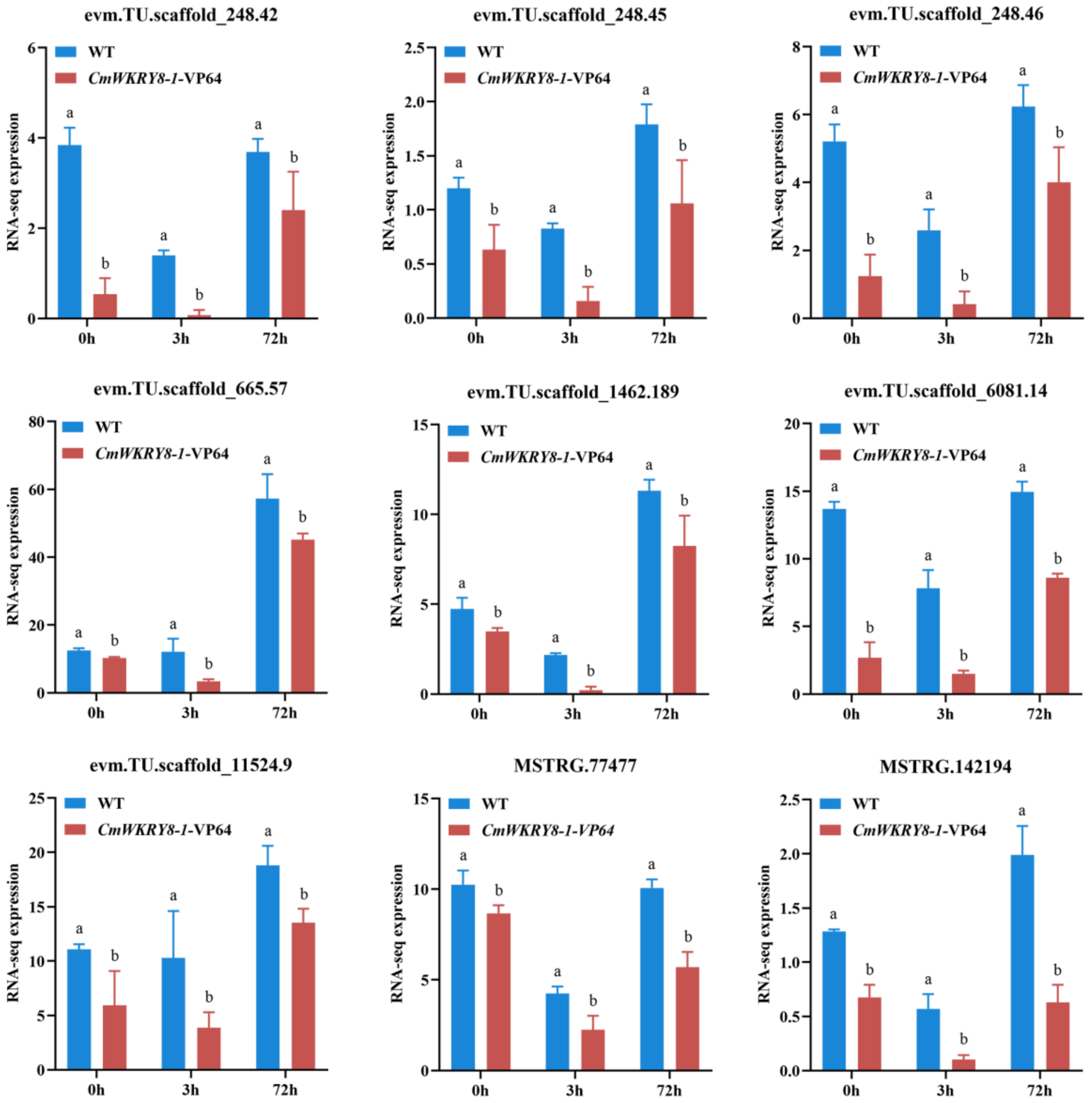

FIGURE S3| Expression levels of DEGs of the SA signalling pathway in the transcriptome of WT and *CmWRKY8-1* transgenic lines at 0h, 3h, and 72h after inoculation treatment.
