# Supplementary material for: Overexpression of CmWRKY8-1–VP64 Fusion Protein Reduces Resistance in Response to Fusarium oxysporum by Modulating the Salicylic Acid Signaling Pathway in Chrysanthemum morifolium
Source: Int J Mol Sci. 2023 Feb 9;24(4):3499. doi: 10.3390/ijms24043499 (PMC9964100; doi:10.3390/ijms24043499)
Supplement: Supplementary file 1 [file ijms-24-03499-s001.zip › Table S1.pdf]

Table. S1. Open reading frame of *CmWRKY8-1*

Gene	Sequence
CmWRKY8-1	ATGAATCTAAACTCATCGAGCAATCATCACTCAGATAG<br>TAATTGTCTGTATAGCAAGTTAAAAAAAGGTACAGAAC<br>AATGTAAAAAGCTTCAAATAGATCTGAATTTGCCTGCA<br>TCTTCTAACGAAACCGAAGAGTTGTCGACTCACAACAT<br>CCCCAACTCTGATGACGAGGCTGTGTCACGGTATGCTG<br>GAACGCTAAATAGGTCAGAATATCCAAGACAATTGAAT<br>GGCAGGCCACATAGTGATGATTCTGACACCAAGGATTC<br>CAATCAAAAAGATGCCTCAAGGAAGAAAAAGAGTTGC<br>AGTAGTAGCATCGTTAAGTGGAAGCAACAAGTGAAAGT<br>GAGTCTGGAGATAGGGCTTGAAGTGCCCCCAAATGATG<br>GTTATATGTGGAGGAAGTATGGTCAAAAGGAAATCCTT<br>AATGCTAAATACCCCAGAGAATATTACAGATGCACCTA<br>TAGAAACACACATGGTTGTTGCGCAACTAAACAAGTTC<br>AAAGGTCTAGTGACGACCCATCTATTTTTGAAATCACTT<br>ACCTTGGAAAACACACTTGTCCCAAACTTTCCAAAACC<br>AACCAATGCTCATCTACTAGTGAATCAGGTTATGGCCTT<br>ATAAATTTCTCAGCTACACCAGAACCTGCATCCAAATCT<br>TCAAGAACCAGTGATCCCTCGTCTGCAACACAATCAGC<br>TTCTACAATTACCAGAAATTCTTTTGAACTCTCAAATTG<br>GCCTCTGAATCATAACTGA
