# Supplementary material for: Overexpression of CmWRKY8-1–VP64 Fusion Protein Reduces Resistance in Response to Fusarium oxysporum by Modulating the Salicylic Acid Signaling Pathway in Chrysanthemum morifolium
Source: Int J Mol Sci. 2023 Feb 9;24(4):3499. doi: 10.3390/ijms24043499 (PMC9964100; doi:10.3390/ijms24043499)
Supplement: Supplementary file 1 [file ijms-24-03499-s001.zip › Table S2.pdf]

Table. S2. All primer sequences used in this experiment

Primer	Sequence
CmWRKY8-1-clone-F	ATGAATCTAAACTCATCGAG
CmWRKY8-1-clone-R	TCAGTTATGATTCAGAGGCC
CmWRKY8-1-qPCR-F	GGTATGCTGGAACGCTAA
CmWRKY8-1-qPCR-F	GGCACTTCAAGCCCTAT
ICS1-qPCR-F	TTGTCTTCGCTTCATCCAACTCCAG
ICS1-qPCR-R	ATTCACTCTCGCCACCACCAAAC
PAL-qPCR-F	CGGTGTTGAGGGTGGATTCTTTGAG
PAL-qPCR-R	AATGGCGGACAAGACTTCTGCTAAC
EDS1-qPCR-F	GCTGTGTAACTTTCGGGTCT
EDS1-qPCR-R	ACGAGGAACAATGTCGTGTT
EDS5-qPCR-F	ATCACACTCACCCACCCTAA
EDS5-qPCR-R	TCGAAGATATTGCGGCGTAG
PBS3-qPCR-F	AGAAAGCTGATGCCAACGAT
PBS3-qPCR-R	CCGGAACAAACTGGCTCATA
EPS1-qPCR-F	GCTGCAAACCGGGATAAGTA
EPS1-qPCR-R	CTCTTGAACCACCAAACCCA
PR1-qPCR-F	CTAGCCATTCTCCACACCAC
PR1-qPCR-R	CACCAGAGTGGACGAGATTG
PR2-qPCR-F	CCAGGGACCGTAGTACAAGA
PR2-qPCR-R	GCCATCCCGTTTCTGATACC
PR5-qPCR-F	GCTGGAGGTGCAGATGGAAA
PR5-qPCR-R	ATCCGTCTTTCACAGCGAGC
EF1α-F	TTTTGGTATCTGGTCCTGGAG
EF1α-R	CCATTCAAGCGACAGACTCA
35S	GACGCACAATCCCACTATCC
