# Supplementary material for: Overexpression of CmWRKY8-1–VP64 Fusion Protein Reduces Resistance in Response to Fusarium oxysporum by Modulating the Salicylic Acid Signaling Pathway in Chrysanthemum morifolium
Source: Int J Mol Sci. 2023 Feb 9;24(4):3499. doi: 10.3390/ijms24043499 (PMC9964100; doi:10.3390/ijms24043499)
Supplement: Supplementary file 1 [file ijms-24-03499-s001.zip › Table S3.pdf]

Table. S3. Chrysanthemum *Fusarium* wilt disease incidence grade index

**The classification of disease severity**	**Symptom description**
0	No disease
1	Only one leaf at the base turns yellow or curls
2	1/3-1/2 of the whole plant leaves become yellow and curled or wilted, the plant is slightly shorter
3	1/2-3/4 leaves of the whole plant become yellow and curled or wilted, leaves fall off
4	Whole plant leaves gum yellow and curled or wilted, or the plant died
